# The Role of Sperm Membrane Potential and Ion Channels in Regulating Sperm Function

**DOI:** 10.3390/ijms24086995

**Published:** 2023-04-10

**Authors:** Francisco M. Pinto, Ainize Odriozola, Luz Candenas, Nerea Subirán

**Affiliations:** 1 Instituto de Investigaciones Químicas, CSIC-University of Sevilla, Avenida Américo Vespucio 49, 41092 Sevilla, Spain; francisco.pinto@iiq.csic.es; 2 Department of Physiology, Faculty of Medicine and Nursery, University of the Basque Country (UPV/EHU), 48940 Bizkaia, Spain; ainize.odriozola@ehu.eus (A.O.); nerea.subiran@ehu.eus (N.S.); 3 Biocruces-Bizkaia Health Research Institute, 48903 Barakaldo, Spain; 4 MEPRO Medical Reproductive Solutions, 20009 San Sebastian, Spain

**Keywords:** Na^+^ channels, K^+^ channels, Ca^2+^ channels, spermatozoa, male infertility

## Abstract

During the last seventy years, studies on mammalian sperm cells have demonstrated the essential role of capacitation, hyperactivation and the acrosome reaction in the acquisition of fertilization ability. These studies revealed the important biochemical and physiological changes that sperm undergo in their travel throughout the female genital tract, including changes in membrane fluidity, the activation of soluble adenylate cyclase, increases in intracellular pH and Ca^2+^ and the development of motility. Sperm are highly polarized cells, with a resting membrane potential of about −40 mV, which must rapidly adapt to the ionic changes occurring through the sperm membrane. This review summarizes the current knowledge about the relationship between variations in the sperm potential membrane, including depolarization and hyperpolarization, and their correlation with changes in sperm motility and capacitation to further lead to the acrosome reaction, a calcium-dependent exocytosis process. We also review the functionality of different ion channels that are present in spermatozoa in order to understand their association with human infertility.

## 1. Introduction

Infertility is defined as the inability to conceive after 12 months or more of regular unprotected intercourse [1]. Currently, this reproductive disease is considered a global health and social problem estimated to affect between 8% and 15% of couples of reproductive age worldwide [2]. It has been reported that the male factor is solely responsible for approximately 20% of infertile couples and contributes to another 30% of couples [3]. During the last seventy years, studies on sperm cells have allowed a great advance in our knowledge about the physiology of spermatozoa and a parallel development of assisted reproduction technology (ART) as well [4,5,6,7,8,9,10,11]. However, the diagnosis of male infertility remains mainly based on semen analysis, and semen parameters alone are insufficient to determine infertility/fertility status. Moreover, the cause of abnormal semen parameters cannot always be identified, and no treatment can be offered to those patients [12]. To improve current infertility diagnosis, it is necessary to have a deeper knowledge about the control of physiological functions of spermatozoa at a cellular and molecular level.

Sperm cells are immature and non-competent immediately after ejaculation [4,5]. In order to obtain fertilization potential, spermatozoa need to undergo capacitation, which implies profound physiological and biochemical changes that occur inside the female genital tract [4]. For this capacitation to take place, spermatozoa must send and receive specific signals from the environment, which must be properly decoded under a precise spatio-temporal regulation that is still not completely unraveled. During their travel, sperm cells suffer an important loss of cholesterol from their membrane, causing an enhancement of membrane fluidity [13]. The increase in extracellular pH, as well as the high bicarbonate concentration in the seminal plasma and in the female genital tract, enable HCO_3_^−^ influx to the sperm cell [14,15,16] and HCO_3_^−^ uptake stimulates the activity of soluble adenylate cyclase (sAC) and the production of cyclic adenosine monophosphate (cAMP), which, in turn, causes the activation of protein kinase A (PKA) [9,17,18,19,20]. PKA induces the phosphorylation of proteins in serine, threonine and, ultimately, in tyrosine (Tyr) residues, which constitutes a mark of sperm capacitation [9,17]. This is followed by a modification of the membrane composition that enables the acrosome reaction (AR) [15,16,21,22]. The control of sperm membrane potential (Em) is essential for all these processes to occur and its regulation is orchestrated by complex signaling pathways that involve activation of a great number of ion transporters, exchangers and ion channels present in the sperm membrane [8,14,15]. Between them, ion channels play a central role due to their rapid response and transport capability [10,11,14,15,23,24,25]. In this review, we analyze the relationships between changes in sperm membrane potential and the functionality of different ion channels that are present in human spermatozoa, in order to better understand their association with sperm function and, consequently, with human fertility.

## 2. Membrane Potential in Spermatozoa

Spermatozoa are highly polarized cells with two clearly differentiated parts, a head and a tail. As occurs in neurons, sperm cells rapidly respond to changes in the environment and the regulation of ion balance across their membrane has been shown to be essential for sperm motility and fertilization capacity. The main ions involved in the acquisition of sperm fertilization ability are Na^+^, K^+^, Ca^2+^, Cl^−^ and H^+^ [26], similar to those which occur in neurons. In addition, the Na^+^/K^+^ ATPase plays an important role in the establishment of the resting Em and in the maintenance of the electrochemical gradients for Na^+^ and K^+^ across the sperm plasma membrane [15,27]. 

In neurons, action potentials play a central role in cell-to-cell communication, assisting the propagation of signals along the neuronal axon toward synaptic boutons situated at the ends of the axon. Changes in Em occur due to the opening of voltage-dependent ion channels present in the cell plasma membrane, which change the polarity of transmembrane potential and generates an action potential [28]. It starts with a temporal Na^+^ permeability increase, producing an influx of this cation and an umbral membrane depolarization (~−55 mV) [29,30]. When Na^+^ is entering the cell (+30 mV), K^+^ permeability increases, but the opening and closing kinetics of K^+^ channels are slower than those of Na^+^ channels. For this reason, when K^+^ gets out of the cell to recover the resting Em, it takes more time to close the K^+^ channels and the K^+^ efflux produces a membrane hyperpolarization (−90 mV). At the time the K^+^ channels are closed, the Na^+^/K^+^ ATPase restores Em [28]. The membrane depolarization induces the activation of Ca^2+^ channels, which are essential for synaptic transmission, causing an increase in Ca^2+^ concentration at the presynaptic membrane. Consequently, neurotransmitters are released to the synaptic cleft and exocytotic vesicles fusion with the postsynaptic membrane to transmit the information to the next neuron [31,32]. 

As happens in neurons, changes in the ion balance across the membrane generate Em adaptations in spermatozoa [33]. During the journey from the testis to the fertilization site, sperm cells encounter different ion concentrations that modulate their ion channel activity, and therefore their Em [15]. External [K^+^] may change from ~39 to 5–8 mM; [Cl^−^] varies from ~27 to 130 mM, and external [Na^+^] goes from 38 to 140 mM between the cauda epididymis and the oviduct [33]. Non-capacitated human spermatozoa maintain a resting Em of around −40 mV [23,34] and ion channels are in a putative inactive state. Once in the female reproductive tract, human sperm are exposed to an increase in [Na^+^], leading to a Na^+^ influx that induces membrane depolarization [35,36], which promote linear sperm motility [36]. In parallel, membrane conductance has lower selectivity for K^+^ than for Na^+^ [37], proving that membrane depolarization prepares spermatozoa for a successful further capacitation [38]. In many species, including humans, sperm capacitation is accompanied by sperm plasma membrane hyperpolarization, with an increase in intracellular net negative charge to ~−60/−70 mV [33,39]. Membrane hyperpolarization, mainly induced by H^+^ and K^+^ currents, is necessary to achieve a successful capacitation in human sperm [23,35,40] and it is a previous event to prepare sperm for the AR [15,40,41]. For these processes to occur, an increase in intracellular [Ca^2+^] is needed, which can be induced by a calcium influx through calcium channels and be accompanied by Ca^2+^ release from intracellular reservoirs [15,34]. As in neurons and other cell types, calcium is needed to induce the exocytosis process known as acrosome reaction, an essential prerequisite for fusion with the oocyte and, therefore, fertilization [34,42].

## 3. Sperm Motility and Depolarization

Sperm maturation and the acquisition of motility initiates in the male genital tract [16,43]. The completion of cell modeling occurs in the epididymis where spermatozoa are coated with many different proteins and other secretory products that induce profound changes in their functional capability, metabolism and biochemistry [44,45,46]. Swimming capacity is also acquired in the epididymis, although due to the acid pH and low HCO_3_^−^ concentration, they do not move actively until their transport to the female genital tract [16,43,46,47]. Prior to ejaculation, sperm cells are mixed with the seminal plasma, a HCO_3_^−^-rich medium formed by secretions from the testis, the epididymis, the prostate, the bulbourethral and the periurethral glands, with a major contribution of the seminal vesicles [43,45,46]. 

Seminal plasma, which constitutes approximately 95% of semen, provides the optimal environment that ensures sperm motility and fertilization ability and is considered as one of the main sources of factors necessary for capacitation [44,45,46]. At the same time, seminal plasma is rich in free cholesterol and Zn^2+^, which act as decapacitation factors and prevent a premature capacitation [15,43,44,45,46]. After ejaculation, seminal plasma proteins form the gelatinous coagulum that inhibits temporarily the initiation of swimming and due to its buffering properties (pH~7.3–8.4) protect spermatozoa from the acidic vaginal milieu (pH~5) [16,43,44,46]. Once in the female genital tract, a small number of spermatozoa are able to enter the cervix after liquefaction of the seminal coagulum, and they continue the travel throughout the female tract, where they are exposed to changes in the ionic environment that enable their motility. 

The fluctuations in ion concentration through the female genital tract induce changes in ion channel permeabilities, which cause Em variations. Remarkably, it has been shown that Na^+^ changes have a great influence on the sperm Em [48,49,50,51]. The importance of this cation is further supported by the essential role played by the Na^+^/K^+^ ATPase, the electrogenic pump which maintains Na^+^ and K^+^ gradients across the cell membrane, in the regulation of sperm function. The Na^+^/K^+^ ATPase is a heteromeric membrane protein composed of two major polypeptides, an α and a β subunit, making a complex that associates with a γ subunit of the FXYD family [27,52]. The α subunit, formed by 10 transmembrane (TM) segments, constitutes the catalytic subunit and contains the binding sites for the cations and ATP, as well as for ouabain, which is a potent inhibitor of the pump activity, while the single membrane-spanning β subunit is necessary for localization of the ATPase to the plasma membrane [52,53]. Four different isoforms of the α subunit (named α1, α2, α3 and α4) and four different β subunits (named β1, β2, β3 and β4) have been cloned and each of them exhibit unique tissue expression profiles [52]. Between them, the α4 subunit is of particular interest, as it has been only detected in the testes and sperm of various mammalian species, being abundantly expressed in the sperm flagellum [27,49,54,55]. In mouse and rat sperm, blockade of the α4 subunit with ouabain impairs sperm motility and hyperactivation, causes sustained membrane depolarization, a decrease in pHi and an increase in [Na^+^]i and [Ca^2+^]i [27,49]. The essential role of the α4 subunit in sperm has been demonstrated by studies in α4 subunit-null mice, which show functional alterations similar to those observed after ouabain treatment and are infertile [27]. Human spermatozoa are exposed to an increase in [Na^+^] in the female reproductive tract, which induces a membrane depolarization caused by Na^+^ influx [35] and promotes linear sperm motility [36]. Sperm membrane depolarization, therefore, can be mediated by activation of Na^+^ channels and, among them, the presence of ENaC channels and of voltage-gated Na^+^ channels (VGSC) has been detected in human spermatozoa (Table 1).

### 3.1. Epithelial Na^+^ Channels (ENaC)

The addition of external Na^+^ produces a depolarization in sperm cells that is potently inhibited by amiloride and its analog EIPA (5-(N-ethyl-N-isopropyl)-amiloride) [15]. ENaC are heteromultimeric Na^+^ selective channels made up of 4 subunits: α, β, γ and δ [63]. They are regulated by intracellular pH and Ca^2+^, extracellular Na^+^, Cl^−^ and phosphorylation and inhibited by amiloride and EIPA [58,64]. These channels play a central role in the electrogenic Na^+^ transport in a variety of tissues and different studies have shown that they are essential for regulation of Em in spermatozoa [15,51,56,57,58]. The presence of three ENaC subunits, α, β and δ have been demonstrated in sperm from different mammalian species including humans, specifically in the flagellar midpiece and principal piece of the sperm (Table 1) [51,56,58,59], and an amiloride-sensitive inward Na^+^ current has been recorded in mouse spermatogenic cells [51]. This suggests a role for ENaC channels in sperm motility, which is confirmed by the fact that blockade of these channels increases sperm motility in asthenozoospermic patients [56]. Na^+^ permeability is involved in the establishment of the sperm resting Em, and it is thought to be reduced during sperm capacitation [50,51]. These channels, therefore, contribute to the regulation of Em in human sperm [58]. 

### 3.2. Voltage-Gated Na^+^ Channels (VGSC)

Veratridine, a highly selective VGSC activator, causes membrane depolarization and increases intracellular Na^+^ in human sperm cells, leading to a concentration-dependent increase in progressive sperm motility [61,65]. Voltage-gated sodium channels (Nav channels) belong to the voltage-gated ion channel (VGC) superfamily and are complex proteins composed of an α and one or more auxiliary β subunits [29,66,67]. The α subunit is a large protein that consist of four homologous domains, each with six transmembrane (TM) segments, and forms the ion-conducting aqueous pore. Nine different Nav α subunits (Nav1.1–Nav1.9) and a tenth, related, voltage-insensitive atypical isoform (Nax) have been cloned in mammals, each of them encoded by a different gene. Four different β subunits, named β1, β2, β3 and β4, are currently known and each of them consist of a single membrane-spanning segment with a large extracellular N-terminal domain and a smaller intracellular tail [25,29,68]. The mRNA of all VGSC, both α and β subunits, are highly expressed in the human testis, at a level comparable to that found in brain tissues [36,60] and, with the exception of the auxiliary subunit β2, they are also present in sperm (Table 1) [36,69]. Immunofluorescence studies have shown that Nav channel proteins are present in human sperm cells and display specific and different sites of localization, with Nav1.2, Nav1.6, Nav1.8 and Nax being predominantly localized in the flagellum, and Nav1.4, Nav1.7 and Nav1.9 in the connecting piece [36,61]. Nav1.8 has also been detected in bull [70] and ovine sperm [71] and its expression in the ovine sperm transcriptome is strongly downregulated under conditions of heat stress [71].

Functional studies have shown that veratridine causes time- and concentration-dependent increases in progressive sperm motility that are reduced in the presence of tetrodotoxin (TTX) or A-803467, a specific Nav1.8 antagonist, suggesting that the effects of veratridine on motility involve activation of different, TTX-sensitive and insensitive Nav channels [61,62,65]. In addition, veratridine does not induce hyperactivation or AR by itself, but is able to inhibit the progesterone-induced AR [61,65]. Using fluorimetry, it was shown that veratridine causes membrane depolarization and increases intracellular Na^+^, but only induces a minor increase in the intracellular Ca^2+^ concentration in capacitated spermatozoa, which is not produced in non-capacitated cells [65]. All these data demonstrate that veratridine does not act by activating Ca^2+^ channels and suggest that VGSC might be involved in the regulation of the basal, linear progressive sperm motility and acquisition of capacitation, avoiding a premature hyperactivation and AR in an inadequate place.

## 4. Sperm Capacitation and Hyperpolarization

Capacitation implies profound physiological and biochemical changes that initiates in the male genital tract and culminates inside the female genital tract [4,5]. At a molecular level, capacitation is associated to: loss of membrane cholesterol [72,73] and modification of other membrane lipids [74]; activation of a cAMP/PKA pathway [9,20]; increase in protein tyrosine phosphorylation and in intracellular pH (pH_i_) [9,75]. The increase in extracellular pH, as well as the high [HCO_3_^−^] in semen and in the female genital tract [14,16,47] enable a rapid HCO_3_^−^ influx to the sperm cell and induces sperm capacitation [15]. HCO_3_^−^ uptake leads to an increase in flagellar beat frequency by stimulating the activity of the soluble adenylyl cyclase ADCY10 (sAC) and the production of cAMP which, in turn, caused a quick activation of protein kinase A (PKA). This kinase induces the phosphorylation of proteins in serine, threonine and, ultimately, in tyrosine residues [9,17,18,76]. The increase in HCO_3_^−^ regulated sAC activity is necessary for sperm intracellular alkalinization and membrane hyperpolarization [9,58,77] and modulates the activation of K^+^ (KSper) and Ca^2+^ (CatSper) channels, events that are all required for sperm capacitation and sperm motility hyperactivation, respectively [20,47,58]. PKA also regulates the activity of the Na^+^/K^+^ ATPase and causes, depending on the cell type, an activation or an inhibition of the pump [52], although the precise effects on human sperm remain unknown. The importance of sAC in male fertility is demonstrated by the observation that men with homozygous mutations in the gene encoding ADCY10 are infertile [78]. In this context, it has recently been shown that the blockade of sAC with the selective inhibitor TDI-10229 reduces the bicarbonate-induced increase in motility (without affecting basal motility) and prevents PKA activity, Tyr phosphorylation, intracellular alkalinization and the AR in mouse and human sperm, further confirming the key role played by sAC in the acquisition of sperm fertilization ability [20]. 

All this process is orchestrated by complex signaling pathways, with an important participation of Em hyperpolarization, which is regulated by different ion fluxes [23,35,40,51,79]. Specifically, there are two ion mechanisms that mainly contribute to sperm Em hyperpolarization: (1) the reduction in Na^+^ permeability and (2) the increase in K^+^ permeability. PKA inhibitors are able to block the capacitation-induced hyperpolarization, proving that, downstream cAMP signaling, hyperpolarization is necessary to prepare spermatozoa for AR [40,41,80]. The increase in cAMP/PKA causes the activation of K^+^ channels and indirectly inhibits ENaCs through a mechanism that involves the activation of the Cystic Fibrosis Transmembrane Conductance Regulator channel (CFRT) [51,58]. 

CFTR is an ATP-gated anion channel that conducts Cl^−^ and HCO_3_^−^. It is expressed at the equatorial segment of the human sperm head [77] and the flagellar midpiece [51] and mutations in the CFTR gene are responsible for infertility and Cystic Fibrosis disease. The activation of CFRT, which are localized with ENaC in the midpiece of sperm flagella [15,51,56], is coupled to inhibition of ENaC, resulting in membrane hyperpolarization. Consistent with this activity, it has been shown that a reduction in [Na^+^]e or blockade of ENaC by amiloride causes membrane hyperpolarization and increases sperm motility in asthenozoospermic patients [51,56,58]. Similarly, inhibition of CFRT significantly reduced capacitation and HCO_3_^−^-associated events [77] and a specific CFRT inhibitor was shown to prevent ZP3-induced AR and sperm–oocyte fusion in humans [81]. 

On the other hand, K^+^ channels participate in the modulation of the intracellular K^+^ concentration and have a major role in determining Em, being particularly important in sperm hyperpolarization [25,82,83]. They are the most diverse class of ion channels and show a great structural diversity (Table 2). Different types of K^+^ channels, including Ca^2+^-activated K^+^ channels of the SLO subfamilies [84,85], voltage-gated K^+^ channels (Kv channels) [86], inwardly rectifying K^+^ channels (Kir channels) [82,87] and two-pore domain K^+^ channels (K2P) [88,89] have been detected in human sperm.

### 4.1. SLO K^+^ Channels

SLO channels, also known as KSper, have a major role in regulating capacitation- induced hyperpolarization in the sperm cells [23,24,83,84,91]. SLO channels belong to the family of Ca^2+^-activated K^+^ channels (KCa) and are composed of a pore-forming α subunit, and three types of auxiliary subunits [25,85,112,113,114] (Table 2). The four known α subunits are named SLO1, SLO2.1, SLO2.2 and SLO3, and among them, SLO1 and SLO3 are abundantly expressed in mammalian spermatozoa [15,83,84,85,90]. Although SLO are members of the 6 TM K^+^ channels, SLO1 and SLO3 have an additional TM domain, named S0, which play an important role in the interaction with auxiliary subunits. They are, therefore, unique K^+^ channels with 7 TM. The functional protein is an homotetramer, formed by the association of four α subunits, leading to a 7 × 4 TM basic structure [85,112,113,115] (Table 2). Three types of auxiliary subunits have been described for SLO channels: β subunits (β1–4), γ subunits (γ1–4), which belong to the family of extracellular leucine-rich-repeat-containing proteins (LRRC), and the recently described LINGO subunits (LINGO 1–4) that belong to the extracellular leucine-rich repeat and immunoglobulin-like (Ig) domains (LRRIG) protein family [85,92,95,98,114]. The functional properties of SLO channels are dramatically altered by the co-assembled auxiliary subunits, their identity and the number of them. Thus, the α subunit tetramer can be surrounded by 0–4 β subunits, 0–4 γ subunits and a still undetermined number of LINGO subunits [85,113,114]. 

SLO1, also known as KCa1.1, BK or Maxi-K channel, is highly expressed in the flagellum of human spermatozoa [40,90] (Table 2). The SLO1 current is activated by two independent physiological stimuli that act synergistically, [Ca^2+^]i and membrane depolarization, but it is insensitive to intracellular alkalinization [85,90,97,112,113]. This large conductance K^+^ channel is regulated by cholesterol and 17β-estradiol (E2), activated by Mg^2+^ and inhibited by progesterone [90,113,114]. On the other hand, the sperm-specific SLO3 or KCa5.1 channel, an evolutionary duplication of SLO1 in mammals, is also abundant in the human sperm flagella. This channel is sensitive to intracellular alkalinization and membrane depolarization and it is inhibited by progesterone [23,40,84,91,114,116]. In mice, the SLO3 channel is responsible for KSper, and deletion of *Kcnu1*, the gene that encode SLO3, inhibited the alkalinization-induced K^+^ current, and caused infertility [23,83,92,93]. Sperm from these mice are unable to swim progressively, to hyperpolarize and to undergo the acrosome reaction. Remarkably, these sperm cannot acrosome react even when exposed to the Ca^2+^ ionophore A23187. These results show the importance of SLO3 channel activation in the capacitation-associated processes necessary for fertilization [92,93].

In contrast, the human KSper current has mixed characteristics, as it shows weak sensitivity to alkalinization, high sensitivity to [Ca^2+^]i, and is inhibited by high concentrations of progesterone [84,90,91,117,118]. Thus, the precise identity of the channel mediating the [Ca^2+^]i-dependent K^+^ outward fluxes that hyperpolarize the human sperm membrane remains undefined. It has been suggested that human KSper can be mediated by SLO1 [90], by both SLO1 and SLO3 [40,83,91] or by a human-specific SLO3 variant [15,24,84,119]. In this context, a recent study described a human case of male infertility in which a homozygous mutation in SLO3 causes severe asthenoteratozoospermia due to acrosome hypoplasia and mitochondrial sheath malformations [94]. Additionally, the differential characteristic of human KSper may be due to association with specific auxiliary subunits, which can produce profound changes in SLO channel gating properties [85,95,99]. For example, auxiliary β subunits alter channel gating in the presence of elevated [Ca^2+^]i while γ subunits affect SLO gating even at low [Ca^2+^]i [114]. Yang et al. (2009) found that all β subunits can be coexpressed with SLO3 in *Xenopus oocytes*, although only β4, both from human and mice, can modify the channel activation kinetics and surface expression [95]. These authors showed that all β subunits mRNAs are present in mouse sperm [95]. It has also been shown that SLO3 binds strongly to γ1 (LRRC26) and γ2 (LRRC52) subunits but interact only weakly with γ3 (LRRC55) and γ4 (LRRC38) [98]. In mice, the γ2 subunit controls the physiological activation of KSper current and, as shown in *Lrrc52* null mice, is critical for fertility [83,99]. Further studies are needed to determine the identity, stoichiometry and influence of the auxiliary subunits that coassemble with SLO subunits in human sperm. 

### 4.2. Voltage-Gated Potassium Channels (Kv)

Kv channels constitute the largest family of K^+^ channels, with 40 members, and belong to the VGC superfamily. The Kv family is formed by 12 subfamilies (Kv1–Kv12) that are widely distributed in a great variety of tissues [113,120]. The pore-forming α subunit is formed by 6 TM, and the functional channel is a homo- or heterotetramer, composed of 4 α subunits, leading to the classical VGC structure of 24 TM segments [113]. Kv channels may also contain auxiliary β subunits (β1–β3), which regulates channel localization and gating properties [113]. Mammalian Kv channels can be modulated by phosphorylation, being activated by Protein Kinase C (PKC) [121] and downregulated by Tyrosine Kinase (TK) activity [122]. Jacob et al. (2000) described the presence of Kv1.3 mRNA in rat testis and showed that it can be modulated in a similar way [86]. Felix et al. (2002) detected a tetraethyl ammonium (TEA)-sensitive current in mouse spermatogenic cells which was attributed to activation of Kv channels, and Kv1.1, Kv1.2 and Kv1.3 were found to be expressed in mouse spermatozoa [100] (Table 2). The presence of Kv1.1 has also been described in bull spermatozoa [101]. Additionally, male mice with a targeted deletion of the Kv6.4 subunit have immotile spermatozoa and, as a consequence, are infertile [103]. Different studies have shown the presence of other Kv channels in human sperm such as Kv1.5 [89,102] and Kv7.1 [104] (Table 2). Kv7.1, also known as KCNQ1 or KvLQT1, is present in the sperm head and flagellum, and its inhibition reduced sperm motility and AR but had no effect on hyperactivation [104]. The auxiliary subunit KCNE1 is also expressed in human sperm, being mainly localized in the tail and neck regions, and co-localize partially with Kv7.1 [89,104].

In humans, Kv inhibitors, such as Pb^2+^, are able to block the progesterone-induced acrosome reaction, supporting a role for these channels in AR [123,124]. These authors provided indirect evidence probing the presence of Kv channels in human sperm, using a biotinylated charybdotoxin probe, and show that these channels are distributed over the sperm head and colocalize with progesterone receptors [86,123,124]. Several Kv isoforms are sensitive to Pb^2+^ and an inverse relationship between fertilization rates and Pb^2+^ concentration in blood and seminal plasma has also been described [123,124]. Therefore, it has been suggested that Kv channels are responsible for the metal ion-related male infertility, in which AR is prevented [123]. 

However, despite the demonstration of the presence of different Kv channels, recent electrophysiological studies have shown that the K^+^ current recorded in sperm cells is mainly if not solely mediated by activation of SLO channels [23,24,92,93] and the precise role of Kv in sperm function remains to be determined.

### 4.3. Inwardly Rectifying K^+^ Channels (Kir Channels)

Inwardly rectifying K^+^ Channels (Kir channels) have also been described in mammalian spermatozoa [87]. Contrary to Kv channels, Kir channels do not activate by depolarization. Kir comprises a variety of K^+^ channels classified in seven different subfamilies which are activated by intracellular mediators [113,120]. These channels are classified, from a functional point of view, in four different groups: (1) K^+^ transport channels, including Kir1.1, Kir4.1, Kir4.2, Kir5.1, and Kir7.1; (2) Classical Kir channels, comprising Kir2.1–Kir2.4 and Kir2.6 channels; (3) G-protein-gated Kir channels (GIRK), including Kir3.1–Kir3.4, and (4) ATP-sensitive K^+^ channels (K_ATP_), comprising Kir6.1 and Kir6.2 [125]. The pore-forming α subunit is composed of two hydrophobic TM domains and the functional channel derives from the association of four subunits, forming homo- or heterotetramers [113,126]. 

Kir channels play an important role in the maintenance of resting Em [108,113,127,128] and their conductance is strictly regulated by intracellular Mg^2+^ and endogenous polyamines, such as spermine, spermidine and putrescine [108,125]. At membrane potentials positive to the equilibrium potential of K^+^ (E_K_), intracellular Mg^2+^ and polyamines block the channel, allowing a small outward current, while at potentials negative to E_K_, Mg^2+^ and polyamines flow into the cell, and the channel unlocking permits a large inward K^+^ current [125]. Kir channels can also be regulated by intracellular pH, Na^+^ or ATP and their conductance augments at higher extracellular K^+^ concentrations [87,107,113,125,129]. Additionally, the component of the plasma membrane phosphatidylinositol 4,5-bisphosphate (PIP2) plays an essential role in Kir channel activation, with each Kir group showing a differential sensitivity to this signaling phospholipid [125].

The presence of Kir channels in mammalian sperm has been poorly studied. Kir3.1 and Kir3.2d (a splice variant that is expressed only in the testis) have been detected in the rat and mouse testis and mouse sperm [100,105,106,107,125], Kir4.1 in the mouse testis [107], Kir7.1 in the rat testis [126] and Kir5.1 has been identified in the rat testis [108,109] and in the head and body of rat and mouse sperm [107,108]. Recent findings suggest that Kir5.1 could participate in the regulation of pHi changes that occur during sperm capacitation [107] (Table 2). Moreover, the deletion of *Kcnj16* in mice, the gene encoding Kir5.1, increases the percentage of sperm with abnormal flagellar morphology and causes subfertility in an age-dependent manner, providing a role for this channel in male infertility [107]. The detection of Kir4.1 in the mouse testis further support a participation of Kir5.1, as this is only functional as a heteromer with Kir4.1 and/or Kir4.2 [125]. 

Within the Kir family, ATP-sensitive potassium channels (K_ATP_) are a group with differential properties from other members of the family which include Kir6.1 and Kir6.2. These channels have a hetero-octameric structure, composed of four preforming K_ATP_ subunits (Kir6.1 and/or Kir6.2, each of them with 2 TM) and a combination of four regulatory sulphonylurea receptor (SUR) subunits (SUR1, SUR2A and/or SUR2B) [82,113,130]. These channels are weak inwardly rectifiers and are regulated by adenine nucleotides, thus coupling cellular metabolism with membrane excitability and Em [82,125]. K_ATP_ are inactivated by intracellular ATP, causing a membrane depolarization, while intracellular ADP activates the channel through interaction with SUR in a Mg^2+^-dependent manner, leading to membrane hyperpolarization [125]. The channels are also regulated by intracellular acidification, which causes activation, and by PIP2 and other phosphatidylinositol phosphates that antagonize the ATP inhibition with the consequent opening of K_ATP_. The activity of K_ATP_ is also controlled by PKC and PKA phosphorylation [82,125].

The K_ATP_ subunits Kir6.2 and SUR2 have been detected using RT-PCR, Western blot, immunohistochemistry and immunofluorescence in epididymal epithelial cells and epididymal spermatozoa from several mammalian species including rats, mice, dogs, cats, cattle and humans, with strong co-localization [111]. Kir6.1, Kir6.2, SUR1, SUR2A and SUR2B are also present in rat and mouse spermatogenic cells and mature sperm [82,110] and there is a co-localization of Kir6.2 with SUR2B in the acrosome of rat spermatids [110] and of Kir6.2 with SUR1 in the post-acrosomal region and flagellar midpiece of mouse spermatozoa [82]. In mouse sperm, K_ATP_ function has been linked to sperm hyperpolarization and AR during capacitation [82]. More studies are needed to clarify the role of K_ATP_, and in general, of Kir channels in human sperm.

### 4.4. K2P Channels

The family of two-pore domain K^+^ channels (K2P) is composed of 15 different subunits (K2P1–7, K2P9–10, K2P12–13 and K2P15–18) [113]. They are responsible for a voltage-independent K^+^-selective leak that is regulated by numerous chemical and physiological stimuli such as pH, mechanical stretch, temperature, membrane phospholipid composition, second messengers or activation of G protein-coupled receptors (GPCRs) [113,131]. These channels play a central role in Em regulation in a wide range of cell types [131]. K2P subunits have a structure unique among K^+^ channels, with 4 TM, and the functional channel is formed by two 2P dimers, leading, in a similar way to other K^+^ channels, to a tetramer-like structure [113]. The K2P family is divided in six subfamilies and, among them, the presence of TREK1 (K2P2), TASK2 (K2P5) and TRAAK (K2P4) has been demonstrated in sperm from nonhuman primates [88]. By using an agonist and an antagonist of TRAAK, Chow et al., found that K2P channels participate in the regulation of several kinetic parameters of sperm movement and accelerates AR [88]. Using RT-PCR, Western blot and immunofluorescence, K2P5 has also been detected in human sperm with a major localization around the neck region [89] (Table 2). 

As occurs with Kv and Kir, electrophysiological studies have failed to identify the presence of a K2P current in sperm cells. However, there is increasing evidence suggesting the participation of K^+^ channels other than SLO in the regulation of sperm function. Brown et al. (2016) described an infertile patient whose sperm showed deficient K^+^ currents but intact genes encoding SLO1 and SLO3 [132] and in pig sperm, the Kv and K2P inhibitor quinine increases progesterone-induced AR more than the specific SLO inhibitor paxilline [133]. The wide expression of different K^+^ channels highlights the need for more studies to elucidate the mechanisms underlying their regulation and their specific role in human sperm fertilizing capacity [104,132,133]. 

## 5. Sperm Capacitation and Alkalinization

Sperm intracellular alkalinization is an essential requisite for acquisition of fertilization ability and sperm cells possess different ion channels and transporters that finely regulate intracellular pH. Between them, the voltage-gated proton channel Hv1 plays an essential role in the regulation of pH in human sperm [24,47,117].

### Voltage-Gated Proton Channels in Sperm 

Regulation of sperm pHi is also fundamental for capacitation. Babcock et al. (1983) suggested that the mechanism for proton efflux from bovine sperm was via a voltage-gated proton channel, based on the fact that the sperm cytosol becomes alkaline upon membrane depolarization [134]. Voltage-gated proton channels (Hv1) play an important role in the regulation of pH in human spermatozoa. A full-length Hv1 and a truncated isoform, lacking 68 amino acids at the N-terminal and named Hv1sper, have been detected in human sperm, although the functional differences between them are unclear [24,47,69,117]. Hv1 is an unusual member of the VGC superfamily [135]. Encoded by the *HVCN1* gene, it was identified in 2006 [136,137] and, until now, its activity has only been demonstrated in human sperm [24,117,138]. In contrast to the high complexity of the rest of the family, which are large proteins with 24 TM segments, Hv1 is formed by two identical dimers, each of them with 2 TM domains, and comprise a voltage sensor -structurally homologous to the voltage-gated sensor domain of the other members of the VGC family, a pore and a gate. The dimers can function autonomously [135,138], but a cooperation between them is required to achieve a correct functionality [135,139]. The opening of these channels can be mediated by depolarization, extracellular alkalinization [139], PKA phosphorylation [76], the endocannabinoid anandamide and by the removal of extracellular zinc [76,117]. When they are in an open state, they mediate a specific outward proton current through the membrane, controlling the intracellular alkalinization of human sperm [117]. Hv1 channels are localized in the principal piece of the human sperm flagellum and their proximity to cation channels such as CatSper [24,140], make them ideally positioned to activate pH-dependent proteins of the axoneme and thus to control sperm motility [117]. However, the specific Hv1 isoform present in humans has not been found in other mammalian species, where the precise mechanisms of regulation of intracellular pH are still unclear.

## 6. Sperm Hyperactivation and Calcium Influx

During capacitation, human sperm acquire hyperactivated motility, necessary to reach the oocyte and undergo AR. Most mammalian sperm, including humans’, undergo two phases during motility acquisition: activation and hyperactivation [10,141,142]. The activation phase initiates upon ejaculation, whereas hyperactivation occurs when capacitation begins [10,128]. Active motility occurs after release from the seminal coagulum, as a result of membrane depolarization. Sperm cells acquire a low amplitude, symmetrical flagellar beat that generates progressive motility. Spermatozoa can then migrate through the female genital tract where they are exposed to an increase in the extracellular [HCO_3_^−^] and [Na^+^] together with a decrease in [K^+^] [14]. This novel milieu stimulates a higher motility acquisition characterized by a high amplitude, asymmetrical beating pattern of the sperm tail, known as hyperactivation [142,143]. Although no direct evidences exist in humans, it is thought that this asymmetrical, whip-like bending of the flagellum, commonly referred to as hyperactivation, is essential for mammalian sperm to overcome the protective vestments of the oocyte [15,22,144]. Studies in mammalian species other than human have shown that spermatozoa that reach the oviductal isthmus bind to epithelial cells forming a reservoir [15,145,146] and hyperactivation permits their release from the isthmus and their progression towards the oviductal ampulla for fertilization of the egg. Calcium is required to initiate and maintain hyperactivated motility [10,147]. In hyperactive spermatozoa, swimming behavior is controlled by the propagation of a Ca^2+^-induced wave that changes the flagellar beat pattern. The increase in intracellular [Ca^2+^] can be induced by a calcium influx through calcium channels and/or by Ca^2+^ release from intracellular reservoirs [48,147,148,149]. Many authors suggest that an initial influx of the cation activates a Ca^2+^-induced Ca^2+^ release (CICR) from sperm intracellular stores, generating intracellular Ca^2+^ oscillations, which would lead to not only hyperactivation but also AR [10,148]. Mitochondria might also play an important role in Ca^2+^ buffering and signaling in sperm cells, shaping the kinetics of Ca^2+^ signals and acting as a sink, in order to buffer cytosolic Ca^2+^ level [11,48,146,149,150]. An increase in [Ca^2+^]i is required not only for initiating but also for maintaining hyperactivated motility [10,146,147,151], which can be mediated by the activation of calcium channels [11,152]. (Table 3). The voltage-dependent CatSper channel has been proposed to be the main calcium influx regulator [14], but several studies have shown that CatSper-null mice are able to acquire hyperactivated motility when calcium is released from internal stores [147,149,153,154], suggesting that other voltage-gated calcium channels (VGCCs) may be also involved in the sperm motility hyperactivation (Table 3).

## 7. Acrosome Reaction and Calcium-Dependent Exocytosis

The acrosome reaction consists of the fusion of the outer acrosomal membrane with the sperm plasma membrane in order to release acrosomal enzymes necessary to penetrate the zona pellucida of the egg. Changes in ion permeability are needed to obtain sperm hyperpolarization, which is essential to induce AR [40,181], pointing out the importance of Em regulation for this process to occur. Reinforcing this idea, it has been described that non-capacitated spermatozoa with depolarized membrane potential are unable to undergo AR [181].

The acrosome is a calcium reservoir, and an IP_3_-mediated calcium release is necessary to complete acrosome exocytosis [42,149]. As several ion channel inhibitors can inhibit AR [182], an initial influx via Ca^2+^ channels may activate the opening of calcium reservoirs in the sperm neck, then cause CICR-induced acrosomal calcium release, which facilitates Ca^2+^-regulated membrane fusion through Soluble NSF Attachment Receptors (SNAREs) located in the acrosomal region [42,147,183,184,185]. The fusion between the outer acrosomal membrane and the overlying plasma membrane, at multiple points, would result in vesiculation and the loss of the fused outer acrosomal membrane/plasma membrane with the subsequent exocytosis of acrosomal granules [13].

### 7.1. CatSper Channels

The cation channel of sperm, CatSper, is a sperm-specific voltage- and pH-dependent calcium channel localized in the principal piece of sperm flagellum [151,162]. CatSper belongs to the family of voltage-gated Ca^2+^ channels and is the main channel responsible for Ca^2+^ influx in mammalian sperm [14,155,156,157,158,159,160,161,162,163,164,165,166,167,168,169,170,171,172,173,174,186,187,188,189,190]. CatSper channels comprise four homologous pore-forming α subunits named CATSPER1, CATSPER2, CATSPER3 and CATSPER4 [15,24,25,151,155,162,166]. Unlike other members of the VGCC family, each CatSper subunit is formed by a single repeat of six TM segments. However, functional activity requires the aggrupation of four monomers of CATSPER1–4 to form the channel pore, resulting in a heterotetramer with a primary structure similar to that of other members of the VGCC family [24,76,156,159]. The channel activity also requires the presence of a large number of auxiliary subunits: the transmembrane proteins CATSPERβ, CATSPERγ CATSPERδ and CATSPERε [156,167,169,171] interact with the adjacent voltage-sensing domain of CATSPER4, 1, 3 and 2, respectively, stabilizing the complex [159] while the cytoplasmic proteins CATSPERζ and EF-hand Ca^2+^-binding domain-containing protein 9 (EFCAB9) form a complex associated with the cytoplasmic mouth of the channel pore, regulating its opening and closing [158,159,171,172]. The CATSPERζ-EFCAB9 complex acts as a dual Ca^2+^ and pH sensor [25,172]. Thus, at low pHi, CATSPERζ-EFCAB9 stabilizes the channel which then remains closed at physiological sperm Em [172,187]. The increase in pH induces Ca^2+^ binding to EFCAB9 causing the dissociation of the CATSPERζ-EFCAB9 complex and permitting the activation of the CatSper channel [76,172]. Very recently, new subunits of this highly complex channel, which has been named CatSpermasome, have been characterized. Lin et al. (2021) and Huang et al. (2023) described three new components in mouse sperm: the transmembrane protein 249 (TMEM249), now named CATSPERθ, essential for the CatSper channel assembly during sperm tail formation; the transmembrane protein 262 (TMEM262), which has been named CATSPERŋ, and the rodent- and testis-specific solute carrier organic anion transporter SCLO6C1 [159,190]. These proteins interact with other auxiliary transmembrane proteins of CatSper (CATSPERŋ with CATSPERβ and SCLO6C1 with CATSPERε) and contribute to the assembly and stability of the CatSper complex [159]. Yang et al. (2022) and Hwang et al. (2022) discovered an additional subunit, the testis-specific protein C2 calcium-dependent domain-containing protein 6 (C2CD6), which has been named CATSPERτ [173,174]. This cytoplasmic protein interacts with CATSPER1–4 subunits and with EFCAB9 and play an essential role in the localization of the CatSpermosome by regulating its targeting to the sperm flagella in developing spermatids [173,174]. All these data show that the specificity and precise identity of the subunits in different mammalian species and the complete structure of the CatSpermosome is still not completely resolved [158,159,173,174]. This highly complex channel is organized in four linear columns within the sperm flagella membrane in both humans and mice, generating a unique longitudinal signaling nanodomain in each flagellar quadrant [158,168,171,172,173]. This spatial arrangement, which is still far from being completely unraveled, is absolutely necessary for Ca^2+^ signaling and sperm fertilization ability.

Activation of CatSper requires membrane depolarization and intracellular alkalinization [152,187]. CatSper can also be indirectly sensitized to depolarization, through Hv1 activation and potentiation of intracellular alkalinization [79,188]. The voltage at which half of the CatSper channels are activated in humans is +85 mV versus +11 mV of mouse CatSper at the same pHi (pHi = 7.5) reflecting the profound differences that exist between CatSper channels of different species, in spite of being highly conserved throughout evolution [24,171,187]. In any case, potassium-induced membrane hyperpolarization has been proposed to induce a reduction on CatSper opening [84], suggesting a reciprocal regulation of potassium induced membrane potential alterations and CatSper in order to prevent a premature AR.

CatSper is also sensitive to different activators which vary between species [15,24,76] and insensitive to GPCRs [189,191]. Progesterone at low concentrations and prostaglandins can change the voltage sensitivity of the channel and activate CatSper in human sperm [115,189,192]. In fact, progesterone-induced Ca^2+^ oscillations in human sperm are generated in the flagellum principal piece by Em-sensitive activity of CatSper [152].

The crucial role of CatSper is corroborated by the important alterations produced in sperm function as a consequence of mutations in any of the subunits that forms this complex channel (Table 3). In mice, congenital ablation of *CatSper1*, *CatSper2*, *CatSper3* or *CatSper4* genes inhibits CatSper currents and sperm hyperactivation and causes infertility, although spermatogenesis and initial motility are unaffected [150,151,155,161,162,168]. Sperm from these null mice are unable to fertilize oocytes surrounded by ZP demonstrating that CatSper are necessary to penetrate the zona pellucida [76]. Similarly, *CatSperd^−/−^* male mice, lacking the gene that encodes CATSPER∂, are infertile and their sperm do not express Catsper channels [156]. On the contrary, *CatSperz^−/−^* and *Efcab9^−/−^* mice lines or the double *CatSperz*/*Efcab9* KO mice show severe subfertility but a functional, although disorganized, channel [158,171,172]. Sperm from either single or double-null males show apparently normal morphology and progressive motility but were unable to hyperactivate [171,172]. The loss of EFCAB9-CATSPERζ largely eliminates the pH-dependent activation of CatSper and impairs its regulation by intracellular Ca^2+^, providing further evidence for the involvement of this dual complex in the modulation of Ca^2+^ influx mechanisms of CatSper channels [158,172]. The strong alterations produced in the architecture of the channel complex by the loss of CATSPERζ-EFCAB9 suggest an additional structural role for these two subunits [158,171,172]. With respect to the new subunit CATSPERτ, *C2cd6^−/−^* mice show a disorganized but functional CatSper channel, with a highly reduced conductance [173,174]. *C2cd6^−/−^* mice have an apparently normal spermatogenesis, and spermatozoa with normal morphology and progressive motility. However, sperm cells from these mice are unable to acquire hyperactivated motility and to fertilize eggs, and are therefore infertile [173,174].

Studies in human patients have also been able to detect several deletions and/or mutations in the genes encoding different CATSPER subunits and their association with infertility [24,157,163,164,165,170] (Table 3).

### 7.2. Voltage-Gated Calcium Channels (Cav)

Although Ca^2+^ influx is proposed to be mainly dependent on CatSper in spermatozoa, it may also be mediated by classic voltage-gated calcium channels, Cav, which are also present in these cells of different species [10,48,193,194]. VGCCs belong to the of VGC superfamily and consist of a pore-containing α subunit, named α1, and one or more auxiliary subunits. The α subunit shows the typical structure of VGC members and is formed by a four repeat of 6 TM segments in a single protein [25,66]. There are ten different α1 subunits and, depending on which of them is present, Cav have different opening characteristics and can be classified into two functional groups: high-voltage activated (HVA) and low-voltage activated (LVA) channels [10,66,194]. HVA channels can be grouped in L-Type (Cav1.1–Cav1.4), P/Q-Type (Cav2.1), N-Type (Cav2.2) and R-Type (Cav2.3) whereas LVA channels comprise T-Type channels (Cav3.1–Cav3.3) [48,66,193,194]. With respect to auxiliary subunits, four α2∂ (α2∂1–α2∂4), four β (β1–β4) and eight different γ subunits are currently known. The number and identity of the associated auxiliary subunits depend on the type of Cav channel [66,195].

VGCCs are expressed in sperm from a wide number of species [25,48,69,175,176,177,178,179,180,193,194]. Among them, Cav1.2, Cav2.1, Cav2.2, Cav2.3, Cav3.1, Cav3.2 and Cav3.3 transcripts have been identified in mature human spermatozoa [160,177] and the presence of the auxiliary subunits α2∂, β1, β3 and β4 has also been reported [180] (Table 3).

Patch clamp studies have not revealed Cav1 or Cav2 currents in spermatogenic cells [10]. In contrast, Cav3 currents have been well documented in spermatogenic cells by patch clamp experiments [10,176], probably because weak depolarizations are needed to open Cav3 channels in comparison to Cav1 and Cav2 isoforms, that need high depolarizations. Electrophysiological recordings have demonstrated a gradual decrease in Cav3 currents during spermiogenesis, which become undetectable in epididymal sperm [24]. The blockade of L-type (Cav1) or T-type (Cav3) channels caused an inhibition of ZP3-induced Ca^2+^ influx, leading to AR [180], suggesting that these channels may be present in an electrophysiologically inactive state and be activated during sperm differentiation and maturation [34]. In fact, Cav3.3 colocalizes with CatSper1 in the principal piece of the human flagellum and this association inhibits the T-type Ca^2+^ current, measured in CATSPER1 and Cav3.3 co-transfected mammalian HEK cells [160]. In addition, several authors have reported that capacitation-induced hyperpolarization and its downstream physiological changes are needed to remove Cav from an inactive state, which generates a voltage-dependent Ca^2+^ influx, proving that Cav channels may play a role in modulating AR [34,194].

## 8. Ion Channel Dysfunction and Associated Pathologies

Several ion channels are involved in the correct functioning of sperm cells and their maturation. Thus, ion channel dysfunctions, as well as membrane potential alterations, have important consequences for fertilization. Mutations in different ion channels could be responsible for Em alterations in infertile patients and several ion channel dysfunctions have been associated with subfertility/infertility and related pathologies. The study of defective ion channels would lead to both, determining the origin of some infertility cases and the comprehension of the role of ion channels. Many studies have reported that depolarized human sperm membrane potential values are associated with lower fertilization rates [41,132] and asthenozoospermia [196]. A depolarized membrane potential can also inhibit the progesterone-induced acrosome reaction [65], and accordingly, more hyperpolarized resting Em (−75 to −35 mV) has been associated with fertile men compared with sperm from infertile men (−35 to −10 mV) [132,196].

Alterations in sodium currents have also been associated with altered semen parameters. Zhang et al., demonstrated that the expression of the Na^+^/H^+^ exchanger sNHE is downregulated in asthenozoospermic men [197]. Moreover, Wang et al. (2003) showed that sNHE-null mice were completely infertile with severely reduced sperm motility [198], which could be partially rescued by raising intracellular pH [24], probing the role of sodium channels in the maintenance of sperm motility.

Several studies in spermatozoa from infertile men have also highlighted the importance of potassium conductance in the achievement of human fertility. Brown et al. (2016) showed that 10% of the IVF and ICSI patients with subfertility had abnormalities in K^+^ conductance, generating a depolarization of the sperm plasma membrane [132]. Depolarization of the plasma membrane has been associated with low fertility rates and several K^+^ channels seem to be involved in this process. For example, SLO3 mutant mice are infertile, and they suffer membrane depolarization during capacitation [92]. Mice with congenital ablation of Kir5.1 are subfertile [107]. In addition, Inanobe et al. (1999) found that Kir3.2d mutant mice are infertile and they propose a role for this channel in spermatogenesis [106]. More studies need to be carried out in human sperm to elucidate the potassium channels involved in this process.

It has been described that *CATSPER1* and *CATSPER2* mutations in human sperm are associated with infertility [157,165]. Semen parameters from CatSper defective men are consistent with asthenoteratozoospermia and, therefore, abnormalities in human CatSper lead to in vitro fertilization failures [199] which may also involve a participation of other voltage-gated calcium channels [160]. Studies in human patients have been able to detect several deletions and/or mutations in different CATSPER subunits, i.e., CATSPER1, CATSPER2, CATSPER3 or CATSPERE and their association with infertility [24,157,163,164,165,170].

The second most common male factor infertility cause is a ductal obstruction or dysfunction, which is produced by CFTR mutations [200]. Defective CFTR exchangers in human sperm alter Cl^−^ and HCO_3_^−^ transport compromising human sperm fertilizing capacity [15,81]. It has recently been reported that there is a functional interaction between CFTR and SLC26A3—a solute carrier of the SCL26 family—which drives sperm capacitation [201]. The role of SLC26A8 has not been characterized in human sperm, but a nonsense mutation in this protein can cause asthenozoospermia [202]. Moreover, mutations in SLC26A3 have been associated with subfertility and asthenozoospermia [203,204].

To fully understand the effect of sperm ion channel dysfunctions and their association with infertility, the role of ion channels and transporters in human sperm has to be deeply studied. With a better comprehension of the ion channel operation system, the description of new infertility etiologies will be elucidated, and idiopathic infertility could be reduced.

## 9. Conclusions

The control of sperm Em is a complex process that involves the activation of a great number of ion channels, transporters and protein receptors present in the sperm surface. Between them, ion channels play a central role in the maintenance of sperm physiology and the achievement of a successful fertilization. The introduction of electrophysiological techniques has permitted a great advance in our knowledge of the role of ion channels, particularly CatSper, KSper and Hv1, and their important participation in different sperm maturation processes. However, although a large, increasing number of ion channels have been detected in spermatozoa, only a few of them have been functionally characterized in electrophysiological studies, probably reflecting the complex nature of this highly polarized cell and the difficulty of performing patch clamp studies in regions other than the flagellum. Important advances have also been produced by using ion-channel-null mice models, or by the finding of mutations in specific human genes and their association with male infertility. However, in many cases, men infertility is not associated with mutations in a certain gene, but is acquired throughout life, due to age, environmental conditions or other unknown factors. It must be considered that, in the particular case of humans, studies are performed in mature spermatozoa, following ejaculation and, in many cases, after a selection of the more motile cells present in the semen sample, thus precluding an analysis of the precise role of different sperm proteins. At this point, a better understanding of the physiological role of sperm ion channel activities appears essential to discover the mechanisms underlying a successful fertilization.

## Figures and Tables

**Table 1:** Na^+^ channels expressed in human sperm cells.

Protein Family	Protein	Gene	Transmembrane Domains (TM)	Detection Methods	References
Epithelial Sodium Channels (ENaC)	α subunit	*SCNN1A*	2TM (heteromultimeric)	Western blot, immunofluorescence, RT-PCR (mouse spermatids), immunohistochemistry (human spermatocytes and spermatids)	[56,57]
	β subunit	*SCNN1B*	2TM (heteromultimeric)	Western blot, immunofluorescence, ELISA	[15,58,59]
	δ subunit *	*Scnn1d*	2TM (heteromultimeric)	RT-PCR (mouse sspermatids), Western blot (mouse sperm), immunofluorescence (mouse sperm)	[57]
Voltage-gated Sodium Channels (Nav)	Pore-forming α subunit				
	Nav1.1	*SCN1A*	24 TM	RT-PCR (low expression)	[36,60]
	Nav1.2	*SCN2A*	24 TM	RT-PCR, immunofluorescence	[36,60]
	Nav1.3	*SCN3A*	24 TM	RT-PCR (low expression)	[36,60]
	Nav1.4	*SCN4A*	24 TM	RT-PCR, immunofluorescence	[36,60]
	Nav1.5	*SCN5A*	24 TM	RT-PCR, immunofluorescence	[36,60]
	Nav1.6	*SCN8A*	24 TM	RT-PCR, immunofluorescence	[36,60]
	Nav1.7	*SCN9A*	24 TM	RT-PCR, immunofluorescence	[36,60]
	Nav1.8	*SCN10A*	24 TM	RT-PCR, Western blot, immunofluorescence, functional studies	[36,60,61,62]
	Nav1.9	*SCN11A*	24 TM	RT-PCR, immunofluorescence	[36]
	Nax	*SCN7A*	24 TM	RT-PCR, immunofluorescence	[36]
	Auxiliary β subunits				
	β1	*SCN1B*	1 TM	RT-PCR	[36]
	β3	*SCN3B*	1 TM	RT-PCR	[36]
	β4	*SCN4B*	1 TM	RT-PCR	[36]

* Channel subunits that have been studied in mammalian sperm but not in humans. The species other than humans in which the subunits have been detected and the stage of sperm or tissue are also indicated. ELISA: enzyme-linked immunosorbent assay.

**Table 2:** K^+^ channels expressed in human sperm cells.

Protein Family	Protein	Gene	Transmembrane Domains (TM)	Detection Methods	References
Ca^2+^-activated K^+^ channels (KCa)	Pore-forming α subunit				
	SLO1 (BK or Maxi-K channel) KCa1.1	*KCNMA1*	7 TM × 4 (homotetramer)	Electrophysiology, immunofluorescence Western blot, RT-PCR	[40,90]
	SLO3 KCa5.1	*KCNU1*	7 TM × 4 (homotetramer)	Electrophysiology, immunofluorescence, Western blot, RT-PCR, human gene mutation (asthenoteratozooospermia), gene deletion (mouse, infertile)	[40,84,91,92,93,94]
	Auxiliary subunits				
	β subunits				
	β1 *	*Kcnmb1*	2 TM	RT-PCR (mouse testis and epididymal sperm)	[95]
	β2 *	*Kcnmb2*	2 TM	RT-PCR (mouse testis and epididymal sperm)	[95]
	β3	*KCNMB3*	2 TM	RT-PCR	[90,95,96]
	β4 *	*Kcnmb4*	2 TM	RT-PCR, coexpression with SLO3 (Xenopus oocytes), gene deletion (mouse, fertile)	[95]
	γ subunits				
	γ1	*LRRC26*	1 TM	RT-PCR (testis)	[97]
	γ2	*LRRC52*	1 TM	RT-PCR (testis), Western blot, coexpression with SLO3, and gene deletion (mouse, infertile)	[97,98,99]
	γ3	*LRRC55*	1 TM	RT-PCR (testis)	[97]
	γ4	LRRC38	1 TM	RT-PCR (testis)	[97]
Voltage-gated K^+^ Channels (K_v_)	Pore-forming α subunit				
	Kv1.1 *	*Kcna1*	6 TM × 4 (homo- or heterotetramer)	RT-PCR (mouse spermatogenic cells), Western blot (bull sperm), immunofluorescence (mouse epididymal sperm, bull sperm)	[100,101]
	Kv1.2 *	*Kcna2*	6 TM × 4 (homo- or heterotetramer	RT-PCR (mouse spermatogenic cells), immunofluorescence (mouse epididymal sperm)	[100]
	Kv1.3 *	*Kcna3*	6 TM × 4 (homo- or heterotetramer	RT-PCR (mouse spermatogenic cells), immunofluorescence (mouse epididymal sperm)	[100]
	Kv1.5	*KCNA5*	6 TM × 4 (homo- or heterotetramer	RT-PCR, immunofluorescence, Western blot, flow cytometry	[89,102]
	Kv6.4 *	*Kcng4*	6 TM × 4 (homo- or heterotetramer	RT-PCR (mouse testis), Western blot Gene deletion (mouse, infertile)	[103]
	Kv7.1	*KCNQ1*	6 TM × 4 (homo- or heterotetramer	RT-PCR, immunofluorescence, Western blot, functional studies	[104]
Inwardly Rectifying K^+^ channels (Kir)	Pore-forming α subunit				
	Kir3.1 (GIRK1) *	*Kcnj3*	2 TM × 4 (homo- or heterotetramer)	RT-PCR (mouse spermatogenic cells, rat testis), Western blot, immunofluorescence (mouse spermatocytes, epididymal sperm)	[100,105,106]
	Kir3.2d (GIRK2) *	*Kcnj6*	2 TM × 4 (homotetramer)	Immunofluorescence (mouse spermatids), Western blot (mouse testis), RT-PCR (mouse testis), gene deletion (mouse, infertile)	[106]
	Kir4.1 *	*Kcnj10*	2 TM × 4 (homo- or heterotetramer)	Immunofluorescence (mouse epididymis)	[107]
	Kir5.1 *	*Kcnj16*	2 TM × 4 (homo- or heterotetramer)	RT-PCR (rat testis, spermatocytes, and spermatozoa), immunofluorescence (rat testis, mouse epididymis and sperm), and gene deletion (mouse, subfertile)	[107,108,109]
	Auxiliary subunit of K_v_ and K_ir_				
	β	*KCNE1*	1 TM	RT-PCR, Western blot, immunofluorescence, coexpression with Kv7.1, flow cytometry	[89,104]
	K_ATP_ subfamily Pore-forming α subunit				
	Kir6.1 *	*Kcnj8*	2 TM × 4 (homo- or heterotetramer)	RT-PCR (mouse spermatids), Western blot (rat testis and spermatids), immunofluorescence (mouse sperm)	[82,110]
	Kir6.2	*KCNJ11*	2 TM × 4 (homo- or eterotetramer)	Immunofluorescence, RT-PCR (rat and mouse epididymis, mouse spermatids), Western blot (rat epididymis, testis, and spermatids)	[82,110,111]
	K_ATP_ auxiliary subunits				
	SUR1 *	*Abcc8*	5 +6 +6 TM	RT-PCR (mouse spermatids, rat testis), immunofluorescence (mouse sperm)	[82,110]
	SUR2A	*ABCC9*	5 +6 +6 TM	Immunofluorescence, RT-PCR (rat and mouse epididymis, mouse spermatids), Western blot (rat epididymis, testis and spermatids)	[82,110,111]
	SUR2B *	*Abcc9*	5 +6 +6 TM	RT-PCR (mouse spermatids), Western blot (rat testis and spermatids), immunofluorescence (mouse sperm)	[82,110]
Two-pore domain K^+^ Channels (K_2P_)	Pore-forming α subunit				
	K2P2 (TREK1) *	*KCNK2*	4 TM × 2 (2 dimers)	Immunofluorescence, Western blot (non-human primates)	[88]
	K2P4 (TRAAK) *	*KCNK4*	4 TM × 2 (2 dimers)	Immunofluorescence, Western blot, functional studies (non-human primates)	[88]
	K2P5 (TASK2)	*KCNK5*	4 TM × 2 (2 dimers)	Immunofluorescence, Western blot (non-human primates), RT-PCR, flow cytometry	[88,89,102]
	K2P9 (TASK3)	*KCNK9*	4 TM × 2 (2 dimers)	Western blot	[102]

* Channel subunits that have been detected in mammalian sperm but not in humans. The species other than humans in which the subunits have been detected and the stage of sperm or tissue are also indicated.

**Table 3:** Ca^2+^ channels expressed in human sperm cells.

Protein Family	Protein	Gene	Transmembrane Domains (TM)	Detection Methods	References
Voltage-gated Ca^2+^ Channels: CatSper	Pore-forming α subunit				
	CATSPER1	*CATSPER1*	6 TM	RT-PCR, electrophysiology, immunofluorescence, 3D STORM imaging, mass spectrometry, cryo-ET, human gene mutation (asthenozoospermia and infertility), functional studies (mouse sperm), cryo-EM and MS (mouse sperm), gene deletion (mouse, infertile)	[151,155,156,157,158,159,160,161]
	CATSPER2	*CATSPER2*	6 TM	RT-PCR, electrophysiology, immunofluorescence, 3D STORM imaging, Western blot, cryo-ET, mass spectrometry, in situ hybridization, human gene deletion (asthenozoospermia and infertility), cryo-EM and MS (mouse sperm), gene deletion (mouse, infertile)	[158,159,160,161,162,163,164,165]
	CATSPER3	*CATSPER3*	6 TM	RT-PCR, electrophysiology, immunofluorescence, 3D STORM imaging, in situ hybridization, cryo-ET, human gene mutation (AR failure and infertility), cryo-EM and MS (mouse sperm), gene deletion (mouse, infertile)	[24,150,158,159,161,166]
	CATSPER4	*CATSPER4*	6 TM	RT-PCR, electrophysiology, immunofluorescence, cryo-ET, in situ hybridization, cryo-EM and MS (mouse sperm), gene deletion (mouse, infertile)	[150,158,159,161,166]
	Auxiliary subunits				
	CATSPERβ	*CATSPERB*	2 TM	Cryo-ET, RT-PCR (testis), Western blot, and cryo-EM and MS (mouse sperm)	[156,158,159,167]
	CATSPERγ	*CATSPERG*	1 TM	Cryo-ET, RT-PCR (mouse testis), Western blot, immunofluorescence, Cryo-EM and MS (mouse sperm)	[156,158,159,168,169]
	CATSPER∂	*CATSPERD*	1 TM	Cryo-ET, RT-PCR (mouse testis), electrophysiology, Western blot, mass spectrometry, immunofluorescence, cryo-EM and MS (mouse sperm), gene deletion (mouse, infertile)	[156,158,159,168]
	CATSPERε	*CATSPERE*	1 TM	Cryo-ET, Western blot, immunofluorescence, human gene mutation (impaired sperm function) RT-PCR (mouse testis), cryo-EM and MS (mouse sperm)	[158,159,168,170,171]
	CATSPERζ	*CATSPERZ*	Intracellular	Cryo-ET, immunofluorescence, Western blot, RT-PCR (mouse testis), cryo-EM and MS (mouse sperm), gene deletion (mouse, subfertile)	[158,159,171,172]
	EFCAB9	*EFCAB9*	Intracellular	Cryo-ET, immunoflurescence, RT-PCR (mouse testis), Western blot (mouse sperm), functional studies (mouse sperm), cryo-EM and MS (mouse sperm), gene deletion (mouse, subfertile)	[158,159,172]
	CATSPERτ	*C2CD6*	Intracellular	Immunofluorescence, Western blot (mouse testis and round spermatids), SIM (mouse spermatids), functional studies (mouse sperm), gene deletion (mouse, infertile)	[173,174]
	CATSPERŋ	*Tmem262/Catsperh*	3 TM	Cryo-EM and MS (mouse sperm)	[159]
Voltage Gated Calcium Channels: Ca_v_	Pore-forming α subunit				
	Cav1.2 (α1C)	*CACNA1C*	24 TM	RT-PCR, Western blot (mouse sperm), immunofluorescence (mouse sperm)	[175,176,177,178]
	Cav2.1 * (α1A)	*Cacna1a*	24 TM	Western blot (mouse sperm), immunofluorescence (mouse sperm)	[175,178]
	Cav2.2 (α1B)	*CACNA1B*	24 TM	RT-PCR, Western blot (mouse sperm), immunofluorescence (mouse sperm)	[177,179]
	Cav2.3 (α1E)	*CACNA1E*	24 TM	RT-PCR, Western blot (mouse sperm), immunofluorescence (mouse sperm)	[175,177,179]
	Cav3.1 (α1G)	*CACNA1G*	24 TM	RT-PCR, immunofluorescence (mouse spermatocytes)	[176,177]
	Cav3.2 (α1H)	*CACNA1H*	24 TM	RT-PCR, immunofluorescence (mouse spermatocytes)	[176,177]
	Cav3.3 (α1I)	*CACNA1I*	24 TM	RT-PCR, Western Blot, immunofluorescence, Co-immunoprecipitation with CATSPER1 and CATSPER2 (HEK cells)	[160,177]
	Auxiliary β subunits				
	β1	*CACNB1*	-Intracellular	RT-PCR, immunofluorescence (mouse sperm)	[178,180]
	β2	*CACNB2*	-Intracellular	RT-PCR, immunofluorescence (mouse spermatogenic cells)	[178,180]
	β3 *	*Cacnb3*	-Intracellular	RT-PCR (mouse sperm), immunofluorescence (mouse sperm)	[178]
	β4	*CACNB4*	-Intracellular	RT-PCR	[178,180]
	Auxiliary α2δ subunit				
	α2δ1	*CACNA2D1*	1 TM	RT-PCR	[180]

* Channel subunits that have been studied in mammalian sperm but not in humans. The species other than humans in which the subunits have been detected and the stage of sperm or tissue are also indicated. Cryo-ET: cryo-electron tomography; Cryo-EM and MS: cryo-electron microscopy and mass spectrometry; SIM: Structed illumination microscopy.
